# The discovery of the first human retrovirus: HTLV-1 and HTLV-2

**DOI:** 10.1186/1742-4690-2-17

**Published:** 2005-03-02

**Authors:** Robert C Gallo

**Affiliations:** 1 Institute of Human Virology University of Maryland Biotechnology Institute 725W, Lombard Street, Baltimore, MD, 21201, USA; 2 Department of Microbiology and Immunology University of Maryland School of Medicine 655 W. Baltimore Street Baltimore, MD 21201, USA

## Abstract

I describe here the history leading up to and including my laboratory's discovery of the first human retrovirus, HTLV-I, and its close relative, HTLV-II. My efforts were inspired by early work showing a retroviral etiology for leukemias in various animals, including non-human primates. My two main approaches were to develop criteria for and methods for detection of viral reverse transcriptase and to identify growth factors that could support the growth of hematopoietic cells. These efforts finally yielded success following the discovery of IL-2 and its use to culture adult T cell lymphoma/leukemia cells.

## Background

After arriving at NIH in 1965 I spent my first year as a young physician caring and treating (mostly unsuccessfully) acute leukemias in children: a vivid experience and one which made me absolute in a decision to be fully involved in laboratory research and not return to clinical medicine. My research interest almost from the very start was in the biology of blood cells, and I focused on comparisons of human leukemic cells with normal leukocytes. This was mainly limited to comparative biochemistry. Specifically, I studied enzymes of pyrimidine nucleoside and nucleotide metabolism, tRNA species and their corresponding amino acyl-tRNA synthetases, and finally DNA polymerases [see refs.[1-5] as examples] – though now this approach would seem to be empirical in the extreme, because we have so many obvious rational things in cancer research today. However, at that time "fishing expeditions" were "where things were at". I hoped to uncover clues that might help us better understand the nature of leukemic cells and also their origin.

### Discovery that human T cells made cytokines ("lymphokines") and early hints of human retroviruses

The main leukemia I worked on was acute lymphocytic leukemias (ALL). After all, these were the most common of the acute leukemias and gram quantities of these cells were available from my clinical colleagues at NCI. Importantly, these were the only leukemias for which reasonably similar normal control cells were available, namely, normal human lymphoblasts. Scientists in Philadelphia had just discovered that a plant lectin, phytohemagglutinin (PHA), could induce human lymphocytes to become activated and go through a mitotic cycle. These normal lymphoblasts looked like ALL cells, but these were days before most of us would or could know of the great complexity of subtypes of lumphocytes. Functional discriminatory assays were barely available and monoclonal antibodies with their capacity to provide surface markers were yet to come. Thus, we did not then sub-classify lymphocytic leukemias. Herb Cooper of NIH had learned how to purify lymphocytes from columns packed with nylon; myeloid cells would adhere, but lymphocytes passed through. Cooper generously provided this technique to me. During this period (1968–1970) I became very impressed by the studies of Leo Sachs in Israel and later also of Don Metcalf in Australia who where showing that, like some lymphocytes, myeloid cells could also be grown in the laboratory but not in liquid culture. Instead, they used the technique previously applied to virus transformed cells of cell growth on a methylcellulose solid surface in the form of cell colonies. However, growth was transient and the amount of cells quite limited, precluding many types of biochemical, molecular biological, and virological experiments. Nonetheless, from this system, Sachs and his colleagues and Metcalf and co-workers made seminal discoveries, including a growth/differentiation factor, granulocyte macrophage colony-stimulating factor (GM-CSF), which was specific for the myeloid lineage. Sachs logically believed the main production of GM-CSF would be from myeloid cells, i.e., a feedback regulation – granulopoietic progenitors proliferated and formed "dead end" granulocytes, which should produce their own granulopoietic factor [see refs. [6,7] for reviews].

Meanwhile, while comparing ALL cells to normal lymphocytes, I decided to test the conditioned medium of the PHA-stimulated normal cells for growth factor. The late Alan Wu had just joined me from the laboratories of Till and McCulloch in Toronto shortly after the publication of their famous paper describing hematopoietic stem cell assays (in mice) for the first time. Alan and Joan Prival, a post-doctoral fellow, joined me in reporting the then surprising finding that lymphocytes (T cells) made GM-CSF [8]. This would be the start of my long involvement with "conditioned medium" from PHA-stimulated lymphocytes. Dane Boggs, F. Ruscetti, and co-workers in Pittsburgh had described the same phenomena at almost exactly the same time. These papers were likely among the first to describe lymphokines (lymphocyte-derived cytokines).

In this period (early 1970s) I began to study animal retroviruses because in several animals these kinds of viruses caused leukemias. Thus, no matter whether human retroviruses (leukemia-causing or otherwise) existed or not, a study of animal retroviruses, especially focused on learning their mechanisms of leukemia causation, might provide insights into the mechanisms involved in human leukemias. However, my co-workers and I also decided to search for human retroviruses, an unpopular goal at this time, considering the decades of attempts and failures. I was, nonetheless, encouraged by discussions with William Jarrett, the Scottish veterinarian who discovered feline leukemia virus, and by the work of the late Howard Temin. Temin, of course, had predicted that retroviruses of animals replicated by having their RNA genome transcribed into a DNA form, which would then integrate into the DNA of the target cell. He referred to this integrated form as provirus, the name given to his theory. In 1970 Temin and his colleague Mizutani, and separately, David Baltimore, gave credence to the theory with their discovery of the DNA polymerase carried by all retroviruses, reverse transcriptase (RT)[9,10]. For me it also meant a convenient, inexpensive, and extremely sensitive assay for a retrovirus. (This would be one of two technologies that would be key for later discoveries of all human retroviruses).

RT forms in virions only upon budding from the cell. Consequently, finding this enzyme in media of cultured cells implied release of retrovirus particles, and finding RT from extracts of cells implied the presence of a cell associated virus particles, as for example, virions associated with the cell surface membrane. We found rare cases of leukemia that scored positive in RT assays. The problem, however, was that RT might be a product of a normal cellular gene. We needed to develop the assay not only as a very sensitive one but also one that would distinguish RT from all of the then known cellular DNA polymerases (alpha, beta, and gamma). This became a major objective [see refs. [5] and [11-14] for examples].

Armed with these RT assays we did find a few cases of adult lymphocytic leukemias with RT showing all the characteristics of RT from a retrovirus (we had by then purified and characterized RT from many different animal retroviruses). We published on the one best characterized in Nature New Biology in 1972 [15]. We believed this was a "footprint" of a human retrovirus, but we failed to isolate virus from this patient. (Though we will never know, it is interesting to speculate whether this young adult had ATL because of some clinical similarities to ATL). We also thought it would attract wide interest and excitement in the field. It did not. It was clear that we had to isolate a replicating virus, one we could study, perpetuate, and give to others.

The obvious and easiest approach to virus isolation was by using cell lines. Cell culture technology had become widely available by the 1960s, and many cell lines from different species were available. The approach is generally to co-culture the primary cells (in our case the leukemic cells) with a wide variety of such lines and hope virus will take in one or more. This, of course, would be after scoring positive in the RT assay. However, by this period, there was increasing antagonism to research directed toward the finding of human tumor viruses and especially of retroviruses. The NCI had created the heavily funded Virus Cancer Program which was under attack for failing to find clear evidence of tumor viruses. Moreover, by the mid-1970s there had been not only decades of failure to find human retroviruses, there had been many false starts by many investigators utilizing the co-culture system that involved cell lines, including one by me. The usual problem was a cross contamination with an animal retrovirus. For this reason I became convinced that we had to find ways to grow primary blood cells, but not with the systems of Sachs and Metcalf. These methylcellulose colonies of leukocytes provided too few cells and growth of these cells were limited in number and in time. When we had our next hint of an RT positive leukemic sample it turned out to be from a patient with a myeloid leukemia, so we searched for a growth factor that would maintain and promote growth of human myeloid leukemic cells in liquid suspension culture. This had not been achieved before. From an early-term (first few weeks) abortion, we obtained some human embryonic cells that produced a factor that led to the first successful routine growth of these human leukemic cells in liquid suspension(16). We called these HL (human leukemia) cells, with a given sequential number of the samples we had studied. One of these cultured cell populations became an immortalized cell line, HL-60. It was the first human leukemic myeloid cell line [17] and like almost all the others, the HL-60 cells showed no evidence of virus. However, one growing human leukemic cell culture (not immortalized) did yield virus, and it was anxiously propagated. Unfortunately, the embryonic factor needed to keep these cells alive and growing was lost when the freezer in which they were stored broke down over a long holiday weekend, which was not recognized for some time. (My first lesson in never storing a divisible valuable all in one place!). This led us to a frantic search to find another source. We screened conditioned medium from a wide variety of cell lines and cell strains, including many more fetal cells – all to no avail.

One approach was to culture many different types of cells from many different tissue sources (including human embryos) for several days, collect the media (conditioned media or CM), and add it to leukocytes from normal human cord blood, samples of human bone marrow, and myeloid leukemic cells. In this period (early mid-1970s), a post-doctoral fellow, Doris Morgan, joined our group and took part in the search. As would be expected, CM from PHA-stimulated lymphocytes was one of the cell sources I asked to be screened. Doris was succeeding in growing cells from human bone marrow, and was intensely nursing them daily for months. But they were lymphocytes, not myeloid cells. It was neither unique nor interesting to grow human B cells. Even at this time Epstein-Barr virus (EBV) immortalized cell lines were well known to grow often from normal blood or a bone marrow mixed cell population. Indeed, they were the only kind of blood cells that could be routinely grown in long-term culture, but analyses of the cells revealed that they were T cells, which at that time had only recently been clearly delineated from B cells by certain functional assays (the E rosette assay, for example). The factor we had found in the PHA-CM was a new growth factor. Francis Ruscetti had then joined our group and carried out a set of experiments that demonstrated this more fully, and we reported these results in 1976–1977[18,19] and they were to be the first reports of what we termed a T cell mitogenic factor, later called TCGF, and finally interleukin-2 (IL-2). The purification was later [20]. IL-2 was among the first well-defined cytokines. The combination of IL-2 growth of T cells with sensitive RT assays would be (and still is) the key to the discoveries of human retroviruses in T cell leukemias and AIDS.

### The debate about the possible existence of human retroviruses

In this same period the pressure against attempts to find human retroviruses intensified. It was not only the prevailing atmosphere of failure but also reasonable scientific arguments. For examples: (1) there was little evidence for leukemia viruses in primates. (2) When retroviruses were found in animals they were not difficult to find. Extensive viremia preceded disease, therefore, if they infected humans, they would be easy to find and would have been discovered much earlier. (3) Human sera in the presence of complement lysed animal retroviruses, thereby providing a rational mechanism for the conclusion that humans were protected.

Finally, there were technical difficulties such as the ability to culture primary human cells (see Table 1).

**Table 1:** Factors that led to consensus that human retroviruses did not exist

1. Failure to discover them after an extensive survey by many investigators in the 1950s, 1960s, and 1970s.
2. Ease of detection in animal models because of extensive virema.
3. Difficulties in growing primary human cells.
4. Results showing human sera with complement lysed animal retroviruses.

We reasoned otherwise. Kawakami and colleagues had just discovered gibbon ape leukemia virus, and linked it to chronic myeloid leukemia in that species [21]. Later, we discovered a variant of that virus which caused T cell leukemia [22]. Bovine leukemia virus (BLV) was discovered [23,24], and it was noted that BLV replicated at very low levels thus putting to rest the notion of "extensive viremia always precedes animal retrovirus induced leukemias". The biased view came from the fact that the earlier small animal models were naturally selected for their utility. Consequently, models in which virus is difficult to detect would be selected against. As for human sera lysing retroviruses, unfortunately those studies were limited to tests of retroviruses from non-primates. Later, we would learn that many primate retroviruses, including the retroviruses of many, are not susceptible.

Our ultimate focus on T cell leukemias was dictated by several factors. First, most animal leukemias caused by retroviruses are lymphocytic leukemias and of these T cell leukemias predominate. Second, the first and to this date only leukemia of non-human primates is caused by a retrovirus [21], and a particular strain of this virus which we isolated caused T cell leukemia [22]. Third, fortune dictated that we would end up focusing on human T cell malignancies because of our discovery of IL-2 which allowed us to grow significant numbers of such cells in many but not all instances (not all T cell leukemias or lymphomas respond to IL-2).

One other development also influenced our continuation of the pursuit of human retroviruses. This was a documented interspecies transmission of a gibbon ape leukemia virus (GaLV) from a pet old world Gibbon ape to a new world Wooly monkey. It was well known that retroviruses could move from one species to another, but in all cases these were very ancient events only discovered by analyses of cellular DNA of many animals. But in this case the event occurred "right before our eyes", giving rise to the virus from the Wooly monkey known as simian sarcoma virus [25]. We felt humans could not be excluded, and indeed later we would learn that the first human retrovirus discovered (HTLV-1) has close relatives among many old world primates and may have arisen from an ancient transmission from monkey to man. A more relevant example, of course, is HIV. There is much evidence that it came into humans as a much more recent infection from African primates (see Table 2).

**Table 2:** Factors encouraging us to continue searching for human retroviruses

1. The discovery of bovine leukemia virus (minimally replicates, difficult to find)
2. Technological advances – A. A sensitive specific assay for a footprint of a retrovirus, namely, reverse transcriptase. B. Capacity to grow significant numbers of primary human T cells in liquid suspension culture giving us access to virus detection and isolation, namely by using IL-2.
3. Discovery of a retrovirus causing leukemias in a species close to man, namely GaLV.
4. A documented example of a retrovirus transmission from one species of primates to another, namely GaLV from a gibbon ape to a wooly monkey [26].
5. Purification and characterization of reverse transcriptase from a patient with an adult lymphocytic leukemia (type unknown) 1972 [15].

### Discoveries of HTLV-1 and HTLV-2

The first detection and isolation of HTLV-1 was in 1979, and the first detection came from the analysis of a T cell line established by J. Minna and A. Gazdar from a patient these clinicians called a cutaneous T cell lymphoma. Alternatively, such patients were also called mycosis fungoides or Sezary T cell leukemia depending upon clinical nuances. Though IL-2 was supplied by us for them to use in their initial culturing of these cells, the cells rapidly immortalized. An outstanding post-doctoral fellow, Bernard Poiesz, carried out RT assays on these cells with positive results, and we soon arranged for electron microscopic analysis of concentrated RT plus cultures and found retrovirus particles. Because putative human retroviruses viruses had been found many times before by several investigators in established cell lines, only to be subsequently shown to be accidental laboratory contaminants, by the late 1970s I was well aware that much more had to be done before this work was presentable. For instance, we had to (1) show that the same virus could be isolated from primary tissue samples of the same patient by culturing primary T cells with IL-2; (2) demonstrate that the virus was novel, i.e., not any of the known animal retroviruses; (3) show it could infect human T cells in vitro; (4) demonstrate specific antibodies to the virus in the serum of the patient; (5) demonstrate that proviral DNA could be found integrated in the DNA of the cells from which the virus was isolated; (6) provide evidence that this was not a one-time affair by showing serological evidence of specific antibodies not only in the patient but in others as well. These results were successfully obtained in 1979–1980 and available by the time we submitted and published our first report in 1980 [27], enabling us to follow quickly with several other essential reports [28-33], also including independent isolates from other patients [29,34]. One of these patients was a black woman from the Caribbean, and the second was a white merchant marine who acknowledged sexual contacts in southern Japan and the Caribbean. These and all subsequent isolates of HTLV-1 in our laboratory were from primary cells cultured with IL-2. After an initial struggle to publish in the J. of Virology, fortunately, we were soon able to publish the original report in PNAS, and this opened the door. It soon became clear that HTLV-1 was specifically associated with adult T cell malignancy (usually CD4+ cells) in which the patients frequently had cutaneous abnormalities and hypercalcemia. Clinicians in the United States had not at that time made any distinction of HTLV-1-associated T cell malignancies from other neoplasms, and as noted above collectively referred to these patients with others (non-HTLV associated) as cutaneous T cell leukemia-lyumphomas. However, a few years earlier Kiyoshi Takatsuki and his co-workers Junji Yodoi and Takashi Uchiyama defined clusters of leukemia in southwest Japan with special clinical features and cellular morphology, which when coupled with the geographic clustering, led him to propose in 1977 that this was a distinct form of leukemia. He named it adult T cell leukemia (ATL) [35].

Two events significantly catalyzed the further development of our work and of our understanding of HTLV-1 and its role in T cell malignancies. The first of these (in the summer of 1980) was information from Drs. Tom Waldmann and H. Uchiyama, who had come to NIH as a visit scientist. They brought to our attention the ATL cluster in Japan so in the fall of 1980 I contacted two Japanese friends, the late Yohei Ito, then Chair of Microbiology at Kyoto University and Tad Aoki for more information and for sera from such patients to test for antibodies to HTLV. This specific clinical entity had been described as early as 1977 by Takatsuki and his co-workers Yodoi and Uchiyama, and was called adult T cell leukemia by him. Aoki and Ito sent sera from such patients to me in 1980, and these sera scored positive for antibodies to HTLV-1. Based on these results Ito organized a small meeting at Lake Miwa outside of Kyoto attended by a few co-workers and myself from the U.S. and Aoki, Ito, and several other Japanese scientists most notably Takatsuki, Y. Hinuma, and T. Miyoshi. The meeting was held in March 1981. Several of my colleagues and I presented our results in detail. This included description of several isolates of HTLV-1, characteristics of purified HTLV-1 p24 as well as reverse transcriptase proteins, evidence of integrated HTLV-1 provirus T cell malignancies and healthy volunteers which provided clear evidence for the linkage of HTLV-1 to certain T cell malignancies, and the positive serological results in Japanese ATL patients. In organizing this meeting the intention of Ito was to foster wide collaboration in Japan with me and my co-workers on this disease. The meeting summary was published in Cancer Research in November 1981 [36].

It was only at the end of the meeting when we were summarizing and planning for this collaboration with the Japanese investigators, that Dr. Yorio Hinuma "announced" he too had a retrovirus. He presented EM pictures of virus particles from a cell line established by Dr. Miyoshi by co-cultivation of ATL cells and normal human T cells. These results of Miyoshi were the first indication of the transforming capability of HTLV-l because the cell line that was immortalized was from the normal donor [37]. Later, my colleague M. Popovic was able to make this a routine, that is, we would show that HTLV-1 could routinely immortalize normal human T cells [34]. It was obvious to all that the virus pictures shown by Hinuma were HTLV-1. By the time of this meeting we had already published a few papers on HTLV-1. Hinuma called his isolate ATLV (adult T cell leukemia virus), but argued against collaboration claiming it was not possible to provide human sera from Japan for "cultural reasons". In June 1982 Hinuma and colleagues published on their isolate of ATLV [38]. After comparative analyses of isolates of ATLV and HTLV were performed we published with Japanese colleagues M. Yoshida, T. Miyoshi and Y. Ito that HTLV-1 and ATLV were the same virus [39]. Consequently, we agreed that the virus name should be HTLV to recognize the priority of our virus work, and the disease would be referred to as ATL in recognition of the Japanese priority in distinguishing this malignancy as a specific identity which had been "lumped" with other T cell leukemias/lymphomas in western countries and elsewhere as cutaneous T cell lymphomas [40]. Yoshida was soon to make many of the major advances in the molecular biology of HTLV-1 but this is another story.

The second meeting of considerable importance was in London chaired by the late hematologist Sir John Dacie and attended by Dacie, Drs. Daniel Catovsky, Robin Weiss, Mel Greaves, and William Jarrett among others from Great Britain and by my collaborator in epidemiological studies, Dr. William Blattner, and myself. It was Catvosky who called for this meeting because he noted that we had found HTLV-1 mainly in African Americans and black persons in the Caribbean and he had found an unusual frequency of adult T cell malignancies in Caribbean immigrants to England. He recognized the similarities of their disease to Takatsuki's ATL. Thus, he postulated they were one and the same disease and HTLV-1 would be present in all. He was right. Promptly, Blattner accelerated his studies in the Caribbean and documented that HTLV-1 was endemic in some islands. He and Guy de Thé of France would then show that this result depended upon the particular tribes in Africa from which the individuals descended.

Some of these experiences would be a precursor of a persistent pattern, i.e., HTLVs are not easy to transmit, remain within families and regions over long periods of time, and have old-world linkage. Ultimately, related viruses would be found in old-world primates and more distantly related viruses in some ungulates. The modes of transmission would soon be forthcoming as sexual contact, blood, and mother to child via breast feeding. Later in 1981 we isolated HTLV-2 from a leukemia described as "a hairy cell T cell leukemia" [41], but this strain is far less pathogenic that HTLV-1. Many of the features of these viruses coupled with CD4 T cell tropism would prove to be remarkably similar to those of the virus about to enter our work, HIV.

A companion article in Retrovirology by Kiyoshi Takatsuki recounts the events surrounding the discovery of the disease, adult T-cell leukemia [41].
